# Investigation of the Effect of Different Seed Flours on Gluten-Free Products: Baton Cake Production, Characterization, and TOPSIS Application

**DOI:** 10.3390/foods13060964

**Published:** 2024-03-21

**Authors:** Tugba Dedebas, Nur Cebi

**Affiliations:** 1 Department of Food Technology, Bolvadin Vocational School, Afyon Kocatepe University, Afyonkarahisar 03300, Turkey; tdebas@aku.edu.tr; 2 Department of Food Engineering, Faculty of Chemical and Metallurgical Engineering, Yildiz Technical University, Istanbul 34210, Turkey

**Keywords:** seed flour, gluten-free, baton cake, texture, sensory, TOPSIS

## Abstract

The present study aims to develop gluten-free product formulations by using different seed flours to determine their effectiveness in gluten-free products. For this purpose, a baton cake model was selected; the cake production process was conducted by adding hemp, okra, mustard, or coriander seed flours with rice flour at a ratio of 25% seed flour to 75% rice flour to prepare the cake batters. The physicochemical, textural, and sensory properties, as well as the baking behaviors of the resulting products, were determined. The TOPSIS method was used in the sensory analyses. With the addition of seed flours, a color change occurred in the inner and crust values of the baton cake samples. It was observed that the hardness value of the baton cake samples increased with the addition of seed flour. In the TOPSIS evaluation, which is a multi-criteria decision-making method, the most preferred product in the free baton cake samples was the cake with hemp seed flour added, while the least preferred product was the control group baton cake. According to the findings, it was concluded that the use of seed flours can be used as an alternative ingredient in the production of gluten-free baton cakes that celiac patients can consume in their diets.

## 1. Introduction

Consumers’ dietary habits have changed due to the emergence of various diseases. Especially in recent times, celiac disease, which arises from the consumption of grain products such as wheat, barley, and rye by people sensitive to these, is one of the most common food-related diseases. Celiac disease is a chronic enteropathy triggered by gluten consumption, and its prevalence has significantly increased in the second half of the 20th century and during the 21st century [1,2]. To date, the only known effective treatment for celiac disease is strict adherence to a gluten-free diet and excluding gluten-containing grain products such as wheat, rye, and barley [3,4]. Therefore, rice flour is widely used in the production of gluten-free foods thanks to its important properties such as resembling wheat flour—in terms of having a white color—lacking a distinct taste, not exhibiting allergic effects, and being easily digestible. Rice flour has a lower nutritional value compared to other popular grains such as corn. Moreover, gluten-free flours such as rice flour have lower nutritional value compared to wheat and negatively affect the texture and volume of the final product, as well as its color, appearance, and taste [5,6,7]. Consequently, seed flours, either alone or in combination, are used in the production and processing of foods, particularly because they are rich in protein, certain B-group vitamins, calcium, iron, copper, magnesium, and dietary fiber [8]. Many studies in the literature investigate the use of various seed flours such as fig, almond, grape, okra, quinoa, and soy as alternative raw materials in the production of products such as gluten-free bread, cookies, and cakes [2,7,9,10,11,12]. Within the scope of this study, the aim was to increase the variety of gluten-free products by using seed flours such as hemp, okra, mustard, and coriander, which are preferred as spices and have a sharper aroma. For this purpose, the effect of using flours obtained from different seeds on the physicochemical and organoleptic properties of gluten-free cakes was determined. In addition, the TOPSIS technique, which is a very stable decision-making technique, was used to make sensory evaluations.

## 2. Materials and Methods

### 2.1. Materials

The rice flour, sugar, eggs, sunflower oil, milk, and baking powder used in the production of the gluten-free baton cakes were purchased from local markets. Hemp, coriander, okra, and mustard seed flours were obtained from Tazemiz company (Tazemiz Ltd., Mersin, Turkey). Seed flours were obtained by grinding oil-free seeds into flour.

### 2.2. Characteristic Properties of Seed Flours

The color values of hemp, coriander, okra, and mustard seed flours were measured using a colorimeter (Konica-Minolta, CR400, Tokyo, Japan), and the results were expressed as CIE L*, a*, and b* color values. The bulk- and tapped-density values of seed flours were calculated by following the method introduced by Aslan Türker et al. (2023) [7]. The Carr index value was calculated using the bulk and tapped densities to describe the stickiness properties of the flours [13]. The water- and oil-holding capacities for seed flours were determined with the method of de Moraes Crizel et al. (2013) [13] as the amount of water and oil bound by the sample per gram in g/mL.

### 2.3. Production of Gluten-Free Baton Cakes

**Figure 1:**
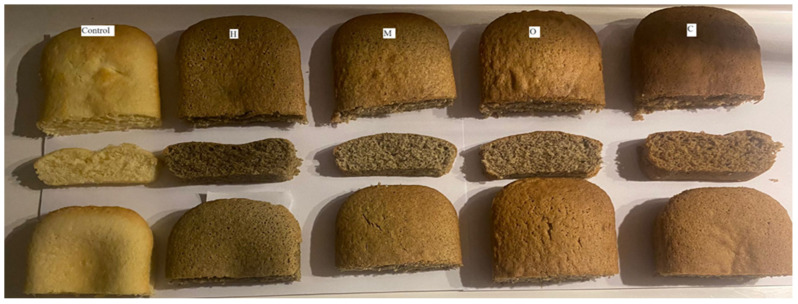
Image of gluten-free baton cake samples.

The quantities of hemp (H), coriander (C), okra (O), and mustard (M) seed flours used in the gluten-free baton cake production were adjusted to 25% of the mixture of seed flour and rice flour, and 5 different cake productions were carried out. The formulations of the flour mixtures used in the cakes’ production are shown in Table 1. For cake production, 110 g of eggs and 200 g of sugar were whisked for a certain period, followed by the addition of 110 mL of vegetable oil, 150 mL of milk, 270 g of flour mixture, and 1.5 g of baking powder as specified in the formulation. In the production of baton cake, initially, eggs and sugar were mixed with a mixer at maximum speed for 5 min. Then, vegetable oil and milk were added and mixed for 1 min. Finally, the gluten-free flour mixture was added. The prepared cake mixtures were baked at 180 °C for 15 min (Figure 1).

### 2.4. Gluten-Free Baton Cakes’ Analyses

#### 2.4.1. Color Values of the Cakes

The color changes in the baton cakes produced using seed flours (hemp, coriander, okra, and mustard) and rice flour were measured using a color measurement device (Konica Minolta, model CR-400, Mississauga, ON, Canada). The crumb and crust colors of baton cake samples were examined and expressed in L* (brightness; 100: white, 0: black), a* (red/green), and b* (yellowness/blueness) values [14]. The total color change (ΔE) was calculated using the formula specified by the ISO/CIE 11664-4:2019 standard: ΔE = [(L*_o_ − L*_control_)^2^ + (a*_o_ − *a_control_*)^2^ + (b*_o_ − *b_control_*)^2^]^1/2^ [15].

#### 2.4.2. Baking Efficiency, Volume, Symmetry, and Uniformity Index Values of the Cakes

The baking efficiency of the cake samples was calculated by dividing the weight of the dough after baking by its weight before baking, and the results were expressed as a percentage. The volume index, symmetry index, and uniformity index values of baton cake samples were determined using the American Association of Cereal Chemists (AACC) Method 10-91 template method [16]. After cooling, the cakes were vertically cut through their centers, and the lengths (mm) of |BB′|, |CC′|, and |DD′| were determined using a millimeter paper, and the results were calculated according to Equation (1):

Volume index (mm) = ǀBB′ǀ + ǀCC′ǀ + ǀDD′ǀ Symmetry index (mm) = 2 × ǀCC′ǀ − ǀBB′ǀ − ǀDD′ǀ Uniformity index (mm) = ǀBB′ǀ − ǀDD′ǀ(1)

#### 2.4.3. Chemical Compositions of the Cakes

The moisture, protein, fat, ash, starch, and cellulose content of baton cake samples produced with hemp, coriander, okra, or mustard seed with rice flour were determined according to AACC (2000) methods, including Method 44-15A for moisture, Method 46-30 for protein, Method 30-25 for fat, Method 08-01 for ash, and Method 992.16 for starch and cellulose [16]. The protein content was calculated using Kjeldahl’s method with a conversion factor of 6.25.

#### 2.4.4. Textural Properties of the Cakes

The textural properties such as hardness (gf), adhesiveness (g.sec), springiness (cm), and cohesiveness of gluten-free cake samples produced with four different seed flours and rice flour were measured using a texture analysis device (T.A.HD Plus Stable Micro Systems, Stable Micro Systems Ltd., Surrey, UK) with a P/36R probe of 36 mm diameter and a 50 N load cell. All measurements were performed in at least five replicates [9]. 

### 2.5. Sensory Properties of the Cakes

A panel of 100 people, 55 women and 45 men, was formed from educated students studying at Afyon Kocatepe University Food Technology Department, ranging in age from 18 to 25. All panelists participating in the research agreed to participate on a voluntary basis. Information about the products was given before the panel. Consumers were asked to evaluate crumb color, porous structure, crust color, taste, smell, aftertaste, and general acceptability of the cake samples using a 1–9-point scale for each criterion. In the evaluation of each criterion for the samples, a rating of 1 represented the lowest score, whereas 9 represented the highest.

### 2.6. TOPSIS

Sensory analysis evaluations were carried out with the TOPSIS technique, which is a multi-criteria decision-making technique. The assumption made in this method is that each attribute has a monotonous increasing or decreasing utility, to simplify the process of locating the ideal and negative ideal solutions.

1. Normalization of the decision matrix (*r_ij_*) can be calculated as (2):
  (2) $$r_{ij}=\frac{a_{ij}}{\sqrt{\sum_{k=1}^{m}a_{kj}^{2}}}$$
2. Decision-maker decides the set of weights (*w*_1_, *w*_2_, *w*_3_, …, *w_N_*), matrix *Y_ij_* as follows (3):
  (3) $$Y_{ij}=\left[\begin{array}{cccc} w_{1}x_{11} & w_{2}x_{12} & \ldots & w_{n}x_{1n}\\ w_{1}x_{21} & w_{2}x_{22} & \ldots & w_{n}x_{2n}\\ . & & & .\\ . & & & .\\ . & & & .\\ w_{1}x_{m1} & w_{2}x_{m2} & \ldots & w_{n}x_{mn} \end{array}\right]$$
3. Calculation of the performance data for n alternatives over *k* criteria. Generally, raw measurements (*r_ij_*) should be standardized (*n_i_*) by the following formulae:
4. Construction of a set of importance weights (*w_k_*) for each criterion: positive (A+) and negative (A−) ideal solution determination (minimum and maximum values);
5. Calculation of the distance of the alternatives from the positive and negative ideal solutions
  (4) $$D_{i}^{*}=\sqrt{\sum_{j=1}^{n}{\left(v_{ij}-v_{j}^{*}\right)}^{2}}$$
  where *v_ij_*, v_j_*, and *v_j_* are the weighted normalized values, and positive and negative ideal solutions, respectively;
6. Determination of the ratio of *R* for each alternative is equal, which is obtained by dividing the distance to the nadir by the sum of the distance to the nadir and the distance to the ideal. Ranking the alternatives using the *R* values and the highest *R*-value is the best alternative [17,18].
  (5) $$R_{i}^{*}=\frac{D_{i}^{-}}{D_{i}^{-}+D_{i}^{*}}$$

### 2.7. Statistical Analysis

At the end of the study, a one-way analysis of variance (ANOVA) and Tukey’s test were conducted using the Minitab 17.0 package program for data evaluation.

## 3. Results and Discussion

### 3.1. Characteristics of Seed Flours

Color is a significant quality attribute that greatly influences consumer preferences [19]. The L*, a*, and b* color values of rice, hemp, coriander, okra, and mustard seed flours are presented in Table 2. While the brightness of flour obtained from mustard seeds was found to be higher when compared to others, the brightness of flour obtained from hemp and coriander seeds was lower. The a* value, indicating redness, was highest in coriander seed flour at 9.52, whereas the redness value of rice flour (0.81) was lower in comparison to others. The b* values, indicating blueness and yellowness, ranged from 9.41 to 29.11. A lower yellowness value was determined for rice flour, while mustard seed flour exhibited a higher yellowness value. There were statistically significant differences between the a* and b* color values of the seed flours (*p* < 0.05). The color values of seed flours obtained by Ofori et al. (2020) after drying seeds of two different genotypes of okra seeds differed from the values found in the present study [20]. Such differences in color attributes are believed to stem from natural color pigments and species’ variations in the structure of seeds [19]. The bulk and tapped densities, as well as the Carr Index values of seed flours, are presented in Table 2. Bulk density is defined as the mass of particles occupying a unit volume of a container. The bulk density of granular solids and powders is largely dependent on particle size, moisture, chemical composition, transportation, and processing operations [14]. The bulk density values of rice, hemp, coriander, okra, and mustard seed flours were determined to be 0.35, 0.65, 0.51, 0.72, and 0.57, respectively. The smoother and more uniform structure of hemp and okra seed flours indicates their smoother nature in comparison to coriander and mustard flours [21,22].

The tapped density values of different seed flours ranged from 0.45 to 0.81 (g/cm^3^). Okra seed flour was found to have the highest tapped density, whereas coriander and mustard seed flours showed lower tapped density values. The significant difference between bulk and tapped densities provides insights into the flowability of powdered products. Coriander and okra seed flours exhibit higher flowability values, while the flowability of mustard seed flour is lower.

The Carr index (%) value is a measure used in the classification of powder flow properties. The flow properties of powders are influenced by particle size and shape, density, electrostatic charge, and moisture [23]. In the Carr index scale, samples with Carr index values between 15 and 20 are defined as having good flow properties, while those between 20 and 35 exhibit intermediate flow behavior. Carr index values for rice, hemp, coriander, okra, and mustard seed flours were determined to be 0.78%, 7.14%, 15.00%, 11.11%, and 3.39%, respectively. Based on the scale, it was determined that coriander and hemp seed flours exhibited better flow properties compared to other seed flours, indicating a high level of flowability.

Water-holding capacity refers to the maximum amount of water a food substance can absorb and retain under formulation conditions; it is also known to be associated with the degree of dryness and porosity [24]. Because it influences the functional and sensory properties of foods, water-holding capacity plays a significant role in the preparation process [9,25]. The water-holding capacity values of the seed flours varied between 1.03 g/mL and 2.03 g/mL (Table 2). It was found that the water-holding capacity of okra seed flour was higher than that of other seed flours. While the water-holding capacity values of hemp and mustard seed flours were similar, statistically significant differences were observed among the samples (*p* < 0.05). Variations in water absorption values among seed flours are thought to arise from differences in their components and their interactions with water. Oil-absorption capacity is important as it enhances the mouthfeel and improves the flavor of food products [26]. As shown in Table 2, hemp (1.50 g/mL) and coriander (1.43 g/mL) seed flours exhibited higher fat absorption capacities compared to okra (0.32 g/mL) and mustard seed (0.94 g/mL) flours.

### 3.2. Color Values of the Gluten-Free Baton Cakes

Among the significant quality factors for consumer preference criteria are the crumb and crust colors of seed-flour-enhanced baton cakes, as shown in Table 3. The L* values, indicating the brightness of both the interior and crust surfaces, were observed to be highest in control samples produced with rice flour, whereas cakes with added seed flours exhibited a decrease in both crust- and crumb-color brightness. The decrease in brightness values of baton cake samples is believed to stem from the original colors of the seed flours [12]. According to the results obtained, coriander seed flour addition shows less shine than other seed flours. The data obtained in this study were consistent with those of Xu et al. (2020) and Tufaro et al. (2022), who produced bread containing okra seed flours and reported producing darker bread [12,27].

The a* value, an indicator of redness and greenness, exhibited a significant increase in color values in both the crust and crumb with the addition of seed flours. This increase is thought to be associated with caramelization or Maillard reactions forming dark brown components. In numerous studies, changes occurring in bakery products produced using different seed flours have been attributed to Maillard reactions [9,11,28,29,30]. Similarly, the b* values of the inner and outer surfaces of baton cake samples ranged from 18.57 to 25.58 and 24.22 to 32.39, respectively. A decrease in yellowness (b*) compared to the control sample was observed with the addition of various seed flours. One study reported a decrease in b* value with the addition of grape seed powder to bread formulation [31]. In another study, it was reported that fruit and vegetable seeds, oilseeds, and plant and herb flours affected the color values of noodles [32]. 

The difference in crust- and crumb-color values of the baton cake samples was expressed as ΔE. The color change in cakes with coriander seeds added was greater than the other seeds.

### 3.3. Baking Efficiency, Uniformity, Symmetry, and Volume Index

Baton cake samples produced using control, hemp, coriander, okra, and mustard seed flours are presented in Table 4 for their baking efficiency, volume index, symmetry index, and uniformity index values. The baking efficiency (%) of baton cake samples varied between 92.50% and 96.00%. It was found that there was no statistically significant difference between the samples (*p >* 0.05). The volume index provides insights into the volume of cakes and is influenced by various factors such as dough consistency, beating speed, beating time, mixing, and baking temperatures [2,33]. The volume index of baton cakes containing control and different seed flours ranged from 50.00 to 71.00 mm (Table 4). While the addition of seed flour resulted in a significant increase in the volume index of cakes, there was no statistical difference among the seeds. Chan et al. (2023) noted variations in volume in chiffon cakes made from rice flour and okra powder mixtures ranging from 2.6% to 10.5% [34]. The disparity in their findings compared to ours may be attributed to the use of 100% seed flour in our baton cakes, resulting in increased volume due to the presence of gums found in okra. Similarly, El-Sayed et al. (2014) stated that cakes made with wheat flour and okra gum as a fat substitute exhibited significantly higher specific volume in comparison to wheat flour cake controls as the level of okra gum increased [35]. The symmetry index value indicates the height differences between the middle and side areas of the cake surface. Thus, a high symmetry index suggests that the middle of the cake is higher than the sides, while a negative or small symmetry indicates a decrease in cake volume after the baking process. This could be interpreted as the cake structure not being stable enough to support its own weight after baking and, consequently, starch gelatinization [28]. The symmetry index values for baton cakes made from control (rice), hemp, coriander, okra, and mustard seed flours were determined as 8, 5, 10, 15.50, and 11, respectively, with a positive symmetry value indicating a raised cake surface. While cakes containing hemp seed flour exhibited lower symmetry values, those with okra seed flour showed more significant expansion [36]; however, with uniformity index values close to zero—indicating homogeneity and cake quality—no statistical differences were found among the analyzed cake samples.

### 3.4. Proximate Analysis of the Cake Samples

The protein (%), moisture (%), ash (%), fat, cellulose, and starch contents of baton cakes produced from rice flour (control), hemp, coriander, okra, and mustard seed flours are presented in Table 5. While the protein content of baton cake samples ranged from 10.12% to 11.16%, it was observed that only the addition of hemp seed flour (11.16%) resulted in an increase in protein content compared to the control sample (10.29%). The addition of other flours did not statistically affect the protein content of the samples [37]. In a study by Mikulec et al. (2019), it was noted that cookies produced with wheat flour containing 20% hemp flour increased from 8.55% to 11.81% [38]. Similarly, the utilization of hemp seed flour in bread samples ranged from 0% to 50%, resulting in an increase in protein content from 11.02% to 19.29%.

The moisture content (%) of baton cakes produced with rice flour (control), hemp, coriander, okra, and mustard seed flours was determined to be 19.96, 17.00, 18.36, 19.49, and 17.10, respectively. The addition of seed flours led to a decrease in moisture content. It was found that the moisture content of cakes with hemp and mustard seed flours was lower compared to others, attributed to the lower water absorption values of these seeds. A similar study by Ertaş and Aslan (2020) indicated a decrease in moisture content in cookie samples where wheat flour was substituted with hemp flour in varying proportions [37]. The moisture content of the control sample produced with rice flour was higher compared to other samples, likely due to the high starch content of rice flour retaining water [11]. 

Baton cakes prepared with different seed flours exhibited a variation in ash content ranging from 3.57% to 6.90%. Generally, an increase in seed flour addition resulted in an increase in the ash content of the cakes, with the highest increase observed in the sample with added hemp seed flour. In a study conducted, it was noted that as the amount of okra seed in composite flour samples increased there was an increase in ash content compared to the control sample—consisting of 100% wheat flour—albeit relatively higher ash content values were reported without the addition of okra seed [39].

The fat content of baton cakes varied between 8.51% and 10.77%, as seen in Table 5. The control sample produced with rice flour has the lowest fat percentage, while the highest fat percentage is observed in samples using hemp and coriander flour. It is presumed that the high fat-binding capacities of hemp and coriander flours contribute to the increased fat content of the resulting product. In a study, it was reported that the fat content in products with added okra seed decreased as the proportion of okra seed flour in the samples increased [39].

The cellulose and starch contents of the baton cake samples containing different seed flours varied between 4.51 and 6.11 and 34.60 and 39.71, respectively. While the cellulose content of control samples produced using rice flour was determined to be the lowest, the starch content was identified as the highest. Pinyo et al. (2024) reported in their study that rice flour has a high starch content [40].

### 3.5. Textural Properties

The textural values of the baton cakes produced with different seed flours and rice flour, including hardness (g), adhesiveness (g.sec), springiness (cm), and cohesiveness, are presented in Table 6. The textural characteristics of the cakes are influenced by factors such as cake volume, moisture content, and the technological properties of the components added to the formulation, such as water and fat binding [33]. The hardness values of baton cakes containing control, hemp, coriander, okra, and mustard seed flours vary, respectively, as 1250.28, 2112.0, 2528, 1947.5, and 2955 gf. With the addition of seed flours, the hardness of the cakes increases, with the softest product being the control sample produced with rice flour, while the incorporation of coriander and mustard seed flours yields firmer products. Similarly, in the study conducted by Konuk Takma et al. (2021), it was noted that the use of fig seed bran flour instead of gluten-free flour increased texture hardness due to its low protein content [11]. Adhesiveness values exhibited behavior similar to hardness among the samples, and the adhesiveness values of baton cake samples obtained from different seed flours showed statistically significant differences. While the most-adhesive product was the control sample, the least-adhesive product was the baton cake produced with M-coded mustard seed flour. Other evaluated textural properties, such as springiness and cohesiveness, ranged between 0.81 and 0.95 cm and 0.49 and 0.57, respectively, with no statistically significant differences observed among the samples (*p* < 0.05). Similarly, although okra flour binds water to gluten-free chiffon cake samples by reducing the evaporation of water during baking, it did not change the elasticity of the test cakes [34]. The spread value of the cake samples indicates their freshness, aerated texture, and elasticity. The springiness values and elasticity of baton cakes produced with seed flours show only a slight increase compared to the control sample [41]. This increase is believed to be due to the increase in protein content in the cakes, as suggested by Bozdogan et al. (2019), who stated that the elasticity of gluten-free cakes increased due to the high protein content of quinoa flour [9].

### 3.6. Sensory Properties

**Figure 2:**
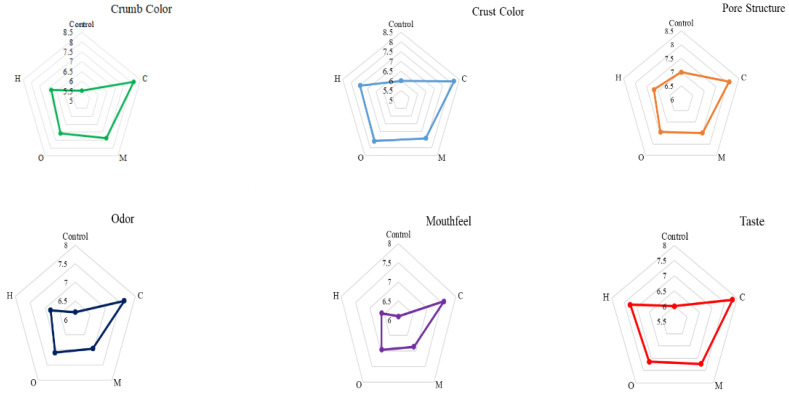
Sensory evaluation of gluten-free baton cakes.

The sensory analysis results of the baton cakes obtained using rice flour and coriander, mustard, okra, or hemp seed flours are shown in Figure 2. Unlike traditional cake production, the gluten-free cake formulation obtained using seed flour is less accepted due to its effects on characteristics such as color and appearance [33]. The crumb color values of the control, C, M, O, and H baton cakes we obtained within the scope of this study were 6.5, 9.12, 8.35, 8.06, and 7.82, respectively; crust color values were 6, 8.18, 7.41, 7.59, and 7.45, respectively. While the crumb- and crust-color values of the baton cake samples were parallel to each other, the cake sample produced with coriander seed flour was liked more by the panelists. The internal pore structure and mouthfeel criteria of the baton cake samples varied between 7 and 8.09 and 6.1 and 7.59, respectively. The internal pore structure and mouthfeel of the cakes produced by adding coriander seed flour were more appreciated than other samples. As seen in Figure 2, the most preferred product was C, with scores of 7.82 and 7.64 in taste and odor values, while the control sample was the least preferred. However, samples labeled as M and H exhibited similar values for the taste and odor criteria. In terms of overall liking, it was observed that gluten-free baton cakes produced with seed flours positively influenced the preferences of panelists, with the most favored baton cakes being ranked as C > M > H > O > Control, respectively. In general, the sensory analysis results show that the addition of seed flour to the cake samples has a positive effect on consumer acceptability.

### 3.7. TOPSIS Analysis

Interpretation of sensory analysis results sometimes yields ambiguous data due to the independent evaluation of each panel question. Therefore, in this context, the utilization of multi-criteria decision-making (MCDM) techniques becomes significant for assessing sensory analysis results. There are studies employing the TOPSIS method for sensory evaluations [42,43,44,45]. In this study, TOPSIS was utilized in the evaluation of gluten-free baton cake samples. The prioritization of panel questions by consumers was determined as pore structure, taste, mouthfeel, odor, crust color, and crumb color. The normalized decision matrix and weighted normalized decision matrix obtained from the analyses used in TOPSIS evaluation are presented in Table 7. The distance and proximity values to the ideal solution, calculated using these values, are provided in Table 8. The preference ranking of gluten-free baton cakes was determined based on the proximity coefficient (R) to the ideal solution. The most preferred product was the cake produced with hemp seed, followed by cakes with coriander, mustard, and okra, respectively. The least-favored product was the control group cake.

## 4. Conclusions

Within this study, nutritionally rich products suitable for celiac patients and people who cannot consume gluten have been developed. In addition, the quality and physical and sensory properties of baton cakes—with seed flours added to support the structure and nutritional content of gluten-free products prepared with rice flour—were examined. Hemp, coriander, okra, and mustard seed flours used in the study enabled the production of cakes with better physical and visual forms than the control group. There was a decrease in the brightness values of the cake samples obtained using seed flours. It was determined that the volume and hardness of the control group cakes produced using rice flour were low. From a sensory perspective, all consumers liked the seed flour cakes more than the control cake. Seed flours, which enrich the loaf cake nutritionally, made significant contributions to the product structure in technological terms. When evaluated from this perspective, our study is valuable because it talks about alternative new products that will meet the needs of the sector. In addition, the fact that seed flours with different flavors can be evaluated in this way and the positive results of the study also support that it is a guiding study in the literature. After this stage, changes in the storage processes, nutritional compositions, and effects on the digestive system of the products obtained can be examined.

## Figures and Tables

**Table 1:** Proportions of seed flours used in cake production.

Samples	Rice Flours (%)	Seed Flours (%)
Control	100	-
H *	75	25
C *	75	25
O *	75	25
M *	75	25

* Hemp (H), coriander (C), okra (O), and mustard (M) seed flours used in gluten-free baton cake.

**Table 2:** Characteristic properties of seed flours.

Seed Flours	Color Properties			Bulk Density (g/cm^3^)	Tapped Density (g/cm^3^)	Carr Index (%)	Water- Holding Capacity (g/mL)	Oil- Holding Capacity (g/mL)
	L*	a*	b*					
Rice	90.8 ^a^ ± 1.69	0.81 ^e^ ± 0.01	9.41 ^e^ ± 1.02	0.35 ^c^ ± 0.00	0.45 ^c^ ± 0.06	0.78 ^e^ ± 0.15	1.03 ^c^ ± 0.10	0.50 ^d^ ± 0.00
Hemp	43.13 ^d^ ± 0.00	4.55 ^c^ ± 0.06	23.05 ^c^ ± 0.15	0.65 ^a^ ± 0.06	0.70 ^a^ ± 0.03	7.14 ^c^ ± 0.10	1.43 ^b^ ± 0.09	1.43 ^a^ ± 0.11
Coriander	42.95 ^d^ ± 0.01	9.52 ^a^ ± 0.23	24.90 ^b^ ± 0.00	0.51 ^b^ ± 0.01	0.60 ^b^ ± 0.03	15.00 ^a^ ± 0.01	1.76 ^ab^ ± 0.34	1.50 ^a^ ± 0.03
Okra	47.68 ^c^ ± 0.03	6.75 ^b^ ± 0.05	18.09 ^d^ ± 1.01	0.72 ^a^ ± 0.05	0.81 ^a^ ± 0.09	11.11 ^b^ ± 0.09	2.30 ^a^ ± 0.09	0.32 ^c^ ± 0.03
Mustard	52.15 ^b^ ± 0.02	2.89 ^d^ ± 0.15	29.11 ^a^ ± 0.05	0.57 ^ab^ ± 0.05	0.59 ^b^ ± 0.00	3.39 ^d^ ± 0.05	1.47 ^b^ ± 0.05	0.94 ^b^ ± 0.09

Different letters in the same column indicate significant differences (*p* < 0.05).

**Table 3:** Crumb- and crust-color values of baton cake samples.

Baton Cake Samples	Crumb Color				Crust Color			
	L*	a*	b*	Δ*E*	L*	a*	b*	Δ*E*
Control	75.25 ^a^ ± 3.04	2.57 ^c^ ± 0.57	25.58 ^a^ ± 0.00	-	70.60 ^a^ ± 0.26	5.87 ^d^ ± 0.12	32.39 ^a^ ± 0.24	-
Hemp	46.53 ^bc^ ± 2.26	4.68 ^bc^ ± 0.79	21.13 ^b^ ± 0.74	29.14	43.52 ^c^ ± 0.33	9.53 ^c^ ± 0.25	24.22 ^e^ ± 0.17	28.52
Coriander	41.01 ^c^ ± 1.44	8.43 ^a^ ± 0.11	18.57 ^c^ ± 0.13	35.44	42.11 ^d^ ± 0.26	14.57 ^a^ ± 0.12	25.54 ^d^ ± 0.25	30.57
Okra	49.53 ^bc^ ± 1.08	5.75 ^b^ ± 0.67	19.04 ^c^ ± 1.03	26.73	49.64 ^b^ ± 0.21	14.47 ^a^ ± 0.40	28.62 ^b^ ± 0.20	22.97
Mustard	53.31 ^b^ ± 2.35	3.67 ^c^ ± 0.07	22.71 ^b^ ± 0.29	22.15	49.22 ^b^ ± 0.31	11.85 ^b^ ± 0.06	27.18 ^c^ ± 0.17	22.80

Different letters in the same column indicate significant differences (*p* < 0.05).

**Table 4:** Physical properties of baton cakes.

Baton Cake Samples	Baking Efficiency (%)	Volume Index (mm)	Symmetry Index (mm)	Uniformity Index (mm)
Control	95.50 ^a^ ± 0.71	50.00 ^b^ ± 1.41	8.00 ^d^ ± 0.00	1.00 ^a^ ± 0.00
H *	96.00 ^a^ ± 0.89	70.00 ^a^ ± 4.24	5.00 ^e^ ± 0.00	3.00 ^a^ ± 1.41
C *	92.50 ^a^ ± 2.12	71.00 ^a^ ± 0.00	10.00 ^c^ ± 0.00	1.50 ^a^ ± 0.71
O *	93.80 ^a^ ± 1.83	68.50 ^a^ ± 0.71	15.50 ^a^ ± 0.71	2.50 ^a^ ± 0.71
M *	93.67 ^a^ ± 1.15	64.00 ^a^ ± 0.00	11.00 ^b^ ± 0.01	1.00 ^a^ ± 0.00

* Hemp (H), coriander (C), okra (O), and mustard (M) seed flours used in gluten-free baton cake; Different letters in the same column indicate significant differences (*p* < 0.05).

**Table 5:** Proximate analysis of baton cakes.

Baton Cake Samples	Protein Content (%)	Moisture (%)	Ash (%)	Fat (%)	Cellulose	Starch
Control	10.29 ^b^ ± 0.03	19.96 ^a^ ± 0.26	3.57 ^d^ ± 0.08	8.51 ^c^ ± 0.25	4.51 ^b^ ± 0.28	39.71 ^a^ ± 0.11
H *	11.16 ^a^ ± 0.17	17.00 ^c^ ± 0.37	6.90 ^a^ ± 4.24	10.65 ^a^ ± 0.09	4.90 ^b^ ± 0.10	34.60 ^b^ ± 0.01
C *	10.12 ^b^ ± 0.16	18.36 ^b^ ± 0.10	4.96 ^b^ ± 0.00	10.77 ^a^ ± 0.01	5.65 ^a^ ± 0.01	35.34 ^b^ ± 0.23
O *	10.52 ^b^ ± 0.13	19.49 ^a^ ± 0.62	4.28 ^c^ ± 0.03	9.37 ^b^ ± 0.39	6.11 ^a^ ± 0.20	35.70 ^b^ ± 0.33
M *	10.37 ^b^ ± 0.07	17.10 ^c^ ± 0.06	5.00 ^b^ ± 0.00	9.47 ^bc^ ± 0.21	5.56 ^a^ ± 0.04	34.96 ^b^ ± 0.49

* Hemp (H), coriander (C), okra (O), and mustard (M) seed flours used in gluten-free baton cake; Different letters in the same column indicate significant differences (*p* < 0.05).

**Table 6:** Texture properties of baton cakes prepared with different seed flours.

Baton Cake Samples	Hardness (gf)	Adhesiveness (g.sec)	Springiness (cm)	Cohesiveness
Control	1250.28 ^b^ ± 12.37	−763.23 ^d^ ± 17.52	0.81 ^a^ ± 0.12	0.50 ^a^ ± 0.03
H *	2112.0 ^b^ ± 74.60	−598.78 ^c^ ± 19.23	0.95 ^a^ ± 0.08	0.55 ^a^ ± 0.03
C *	2528 ^ab^ ± 341	−423.84 ^b^ ± 29.58	0.90 ^a^ ± 0.02	0.49 ^a^ ± 0.02
O *	1947.5 ^bc^ ± 19.0	−655.43 ^d^ ± 30.00	0.88 ^a^ ± 0.06	0.57 ^a^ ± 0.03
M *	2955 ^a^ ± 185	−260.17 ^a^ ± 23.22	0.87 ^a^ ± 0.02	0.54 ^a^ ± 0.00

* Hemp (H), coriander (C), okra (O), and mustard (M) seed flours used in gluten-free baton cake; Different letters in the same column indicate significant differences (*p* < 0.05).

**Table 7:** The decision matrix and normalized decision matrix for TOPSİS application.

**Normalized Decision Matrix**						
**Seed Flours**	**Pore Structure**	**Taste**	**Mouthfeel**	**Odor**	**Crust Color**	**Crumb Color**
Control	0.188	0.169	0.179	0.179	0.164	0.158
C *	0.217	0.221	0.223	0.220	0.223	0.233
M *	0.201	0.204	0.201	0.200	0.202	0.211
O *	0.200	0.201	0.204	0.204	0.207	0.203
H *	0.193	0.195	0.177	0.183	0.200	0.183
**Weighted Normalized Decision Matrix**						
**Seed Flours**	**Pore Structure**	**Taste**	**Mouthfeel**	**Odor**	**Crust Color**	**Crumb Color**
Control	0.063	0.044	0.033	0.020	0.012	0.006
C *	0.072	0.057	0.041	0.024	0.017	0.009
M *	0.067	0.053	0.037	0.022	0.015	0.008
O *	0.067	0.052	0.038	0.023	0.015	0.008
H *	0.064	0.065	0.059	0.061	0.067	0.061

* Hemp (H), coriander (C), okra (O), and mustard (M) seed flours used in gluten-free baton cake.

**Table 8:** Distance from positive (D+), negative (D−), and ratio values of each alternative using TOPSIS techniques.

Alternatives	*D*+	*D*−	*R*	Order of Preference
Control	0.060	0.000	0.000	5
C *	0.047	0.020	0.296	2
M *	0.052	0.012	0.183	3
O *	0.052	0.011	0.180	4
H *	0.008	0.094	0.920	1

* Hemp (H), coriander (C), okra (O), and mustard (M) seed flours used in gluten-free baton cake.

## Data Availability

The original contributions presented in the study are included in the article, further inquiries can be directed to the corresponding author.
